# Cognitive Dissonance Resolution Is Related to Episodic Memory

**DOI:** 10.1371/journal.pone.0108579

**Published:** 2014-09-29

**Authors:** Moti Salti, Imen El Karoui, Mathurin Maillet, Lionel Naccache

**Affiliations:** 1 INSERM, U 1127, F-75013, Paris, France; 2 CNRS, UMR 7225, F-75013, Paris, France; 3 Sorbonne Universités, UPMC Univ Paris 06, UMRS 1127, F-75013, Paris, France; 4 Institut du Cerveau et de la Moelle épinière, ICM, PICNIC Lab, F-75013, Paris, France; 5 Ben-Gurion University of the Negev, Department of Brain and Cognitive Science, Beer-Sheva, Israel; 6 AP-HP, Groupe hospitalier Pitié-Salpêtrière, Department of Neurophysiology, CHU Pitié-Salpétrière, Paris, France; 7 AP-HP, Groupe hospitalier Pitié-Salpêtrière, Department of Neurology, CHU Pitié-Salpétrière, Paris, France; 8 Sorbonne Universités, UPMC Univ Paris 06, Faculté de Médecine Pitié-Salpêtrière, Paris, France; Brain and Spine Institute (ICM), France

## Abstract

The notion that our past choices affect our future behavior is certainly one of the most influential concepts of social psychology since its first experimental report in the 50 s, and its initial theorization by Festinger within the “cognitive dissonance” framework. Using the free choice paradigm (FCP), it was shown that choosing between two similarly rated items made subjects reevaluate the chosen items as more attractive and the rejected items as less attractive. However, in 2010 a major work by Chen and Risen revealed a severe statistical flaw casting doubt on most previous studies. Izuma and colleagues (2010) supplemented the traditional FCP with original control conditions and concluded that the effect observed could not be solely attributed to this methodological flaw. In the present work we aimed at establishing the existence of genuine choice-induced preference change and characterizing this effect. To do so, we replicated Izuma et al.’ study and added a new important control condition which was absent from the original study. Moreover, we added a memory test in order to measure the possible relation between episodic memory of choices and observed behavioral effects. In two experiments we provide experimental evidence supporting genuine choice-induced preference change obtained with FCP. We also contribute to the understanding of the phenomenon by showing that choice-induced preference change effects are strongly correlated with episodic memory.

## Introduction

In 1957, Festinger coined the expression ‘cognitive dissonance’ to convey a general theory postulating that individuals strive to decrease the discomfort generated by conflicting cognitions by modifying one’s cognitions [1]. Since then, this concept has intensively stimulated empirical and theoretical research in social psychology, and it has been so widely popularized that it is frequently used in mass-medias. From an experimental perspective, cognitive dissonance is usually tested through the five following paradigms: induced-compliance [2], belief-disconfirmation [3], effort justification [4], misattribution [5] and free-choice paradigms [6]. The free-choice paradigm (FCP) originated a central claim of cognitive dissonance theory, namely, that our past choices affect our future behaviors, preferences and beliefs.

### An intense theoretical debate about the Free-Choice Paradigm (FCP)

A typical FCP experiment is composed of three blocks. First, subjects rank or rate items according to their desirability (e.g.: food items, car models, holiday destinations, etc…). Ranking and ratings are used interchangeably in FCP paradigms; from this point on we will use only the latter. Second, they are engaged in a forced-choice task during which they have to choose between two closely rated items. Finally, they perform a second rating on the same items. Choice-induced preference change is defined by a tendency to increase ratings of chosen items, and to decrease those of rejected items. This ‘spreading of alternatives’, or ‘spread’, is the hallmark of the phenomenon, and has been considered as diagnostic of preference change. This paradigm has been extensively used during the last decades. However, there is still an intense theoretical debate about the precise mechanisms subtending choice-induced preference change. Schematically, the literature contains two main and opposite types of theoretical accounts.

On the one hand, many different models call for high-level cognitive, self-related (see [7]) or metacognitive top-down accounts of choice-induced preference change. This class of models includes the original Festinger’s model of cognitive dissonance theory [1], as well as other reformulations of it (for extensive review see [8]). Cognitive dissonance postulates the existence of a succession of executive stages triggered by the choice: cognitive conflict generation, detection, monitoring, and resolution. Similarly, other models departing from cognitive dissonance theory share this high-level view of choice-induced preference change. Self-consistency theory proposes that the spread is driven by the need for subjective consistency between previous actions (choice) and current explicit ratings [9], [10]. Self-perception model interprets the spread as reflecting the dynamics of an internal model of the self who is trying to account for his own behavior in the absence of any direct access to an encapsulated value system [11]. Recent neuroimaging findings implicating regions of the executive network such as the anterior cingulate cortex (ACC) strengthen this class of models [12].

On the other hand, other empirical studies suggest that choice-induced preference change is a low-level process which may occur unconsciously and independently from episodic memory and executive control. This conception of choice-induced preference change is supported by the report of spread in amnesic patients [13], in normal controls with very long delays between the two ratings [14], in young infants and even in Capucin monkeys [7], [15]. In the same line, findings of choice-induced preference change correlates in the activity of sub-cortical regions included in the motivation network, such as striatal regions [16], [17] seem congruent with this conception. Indeed, finding a correlate of the spread in the neural value system supports that it is a direct marker of value modifications.

### A statistical artefact potentially flawing most FCP studies

Within this scientific debate, Chen and Risen reported in 2010 that spread could be observed in the FCP even under conditions of stable preferences [18]. Their claim relied on two assumptions. First, they presumed that ratings are noisy measures of subjects’ preferences. Second they suggested that subjects’ choices give additional information about their preferences. Accordingly, if two items, A and B, were similarly rated by a subject, and he chooses A over B, then the most probable account would be that item A is actually preferred over item B, and that initial identical ratings corresponded respectively to under-estimation and over-estimation of genuine preferences for A and B. Therefore, as a result of regression to the mean, one could expect an increase of rating for item A and a decrease of rating for item B during the second rating, without any change in preferences for these items. In other terms, spread is inherent to the FCP design. This devastating theoretical claim was experimentally confirmed by a clever design adding to the classical ‘rating-choice-rating’ (RCR) design, a new ‘rating-rating-choice’ (RRC) sequence. Chen and Risen showed that a spread could be obtained even with this latter sequence, in which choices were made after the second rating. Crucially, in their study RRC and RCR spreads did not differ, suggesting that genuine choice-induced preference change is either inexistent or in any case much smaller than initially reported since 1956. Izuma and Murayama ran simulations of the FCP under Chen and Risen’ assumptions, and confirmed this statistical flaw [19]. They recommended that any attempt to demonstrate a choice-induced preference change should control for it. Since Chen and Risen’ major study, the inclusion of such a RRC sequence in experiments using the FCP seems to be a prerequisite for interpreting any spread as an evidence of genuine choice-induced preference change. It is central to note that most experimental studies using the FCP performed before 2010 did not control for this major artefact. The first studies controlling for it revealed much smaller effect sizes than in the pre-2010 original studies [19]. Therefore, the mentioned theoretical debate remains open given the fragility of previous empirical findings.

In response to Chen and Risen’ criticism, several attempts were made to validate the spreading of alternatives obtained with FCP. Alós-Ferrer & Shi formally refuted the mathematical model presented by Chen and Risen but agreed that on some occasions a positive spread could be observed in the absence of any preference change, and confirmed that ‘the fact that expected spreading for specific rating distances and model specifications might be non-zero makes improved experimental design very valuable’ [20]. Moreover, a few studies have attempted to control for this artifact and still observed choice-induced preference change effects. In particular, Johansson and colleagues (2013) used the choice blindness paradigm to manipulate choices and found a spread effect [21]. Sharot et al. (2012) used a RCR/RRC design to probe very long-lasting effects (>2.5 years). They found a significantly higher spread for RCR than for RRC, but by definition the choice-rating delays were highly asymmetrical between the RCR condition (both rating-choice delays were short) and the RRC condition (the delay between the first rating and the choice was very long>2.5 years). This asymmetry may raise an issue to interpret spread values. Three other recent studies used an ingenious blind choice condition preventing subjects’ choices from revealing more information about their real preferences [7], [22], [23]. However, these results are not immune from criticism. Indeed, the methodology used by Egan et al. (2010) to test infants and monkeys has been put into question. Indeed, in infants, attitude change was estimated by a second blind choice, which thus does not reflect preferences. Moreover, in monkeys, attitude change was assessed by 10 open choices, following the critical blind choice. But according to cognitive dissonance theory, these choices should influenced each other in turn, so the results are hard to interpret (see [19], [24], [25] for detailed reviews of this study). Moreover, Sharot et al.’s study (2010) [22] reported a significant spread only for chosen items while the canonical spread was marginal (p = 0.1).

### Focus on the Izuma et al. (2010) study using a RCR and RRC design

Finally an interesting study by Izuma et al. (2010) used the design recommended by Chen & Risen and reported a significantly larger spread in the RCR condition than in the RRC condition. Interestingly, this work is both cited by proponents of the ‘high-level’ [26], [27] (however none of these two studies properly controlled for the Chen & Risen’s artifact) and of the ‘low-level’ views (e.g.: [14]). A key reason for this ambiguity is to be found in the detail of the design used by Izuma and colleagues.

Indeed, during the second rating of the RCR sequence, each item was labeled with a reminder of the choice the subject had previously made on that item (e.g.: '*you rejected it*’). Obviously, such a reminder was delivered only in the RCR sequence, given that in the RRC sequence the choice was made after the second rating. As a consequence, Izuma et al. did not contrast RCR with RRC conditions, but they rather compared ‘RCR+explicit choice reminder’ versus RRC condition. The probable reason for not choosing the minimal contrast (RCR vs RRC) was to maximize chances of observing choice-induced preference change by capitalizing on all possible cognitive mechanisms (high and low levels). As such, Izuma et al. study succeeded in providing a significant behavioral effect. However, the asymmetry between RCR and RRC conditions makes it simply impossible to disentangle between these distinct cognitive mechanisms.

In the present work we replicated Izuma et al. study and added a strict RCR (without reminder cue) condition. Moreover, we added a memory test in order to measure the possible relation between episodic memory of choices and observed behavioral effects. In a set of two different experiments we reveal a novel and strong correlation between episodic memory of choices and choice-induced preference change.

## Experiment 1

### Methods

#### Ethics Statement

These experiments have been approved by the Pitié-Salpêtrière ethical committee. All the 65 subjects gave their written informed consents, and were paid 10 Euros to participate in the experiment. All investigations were conducted according to the principles expressed in the Declaration of Helsinki.

#### Participants

Twenty-six subjects were included in the ‘Reminder’ group (17 women; age M = 25.3 years old, SD = 5.6; 92% right-handed), and 25 in the ‘No reminder’ group (16 women, age M = 26.4, SD = 5.2; 96% right-handed). They reported normal, or corrected-to-normal, visual acuity. Data from three participants were excluded (2 from the ‘Reminder’ group, and 1 from the ‘No reminder’). One dataset was not saved due to technical failure. The remaining 2 excluded participants essentially used the highest rating values within the 8-values scale in rating 1 (rating 1 median = 8), with a ceiling effect biasing the possible rating changes only to decreased values.

#### Stimuli

Stimuli were 120 colored images of potential vacation destinations. Image subtended 5.3° of visual field. Destination name was printed (font size = 30) below the image. In ‘Rating’ blocks, one image appeared in the center of the screen in each trial, whereas in ‘Choice’ blocks, two targets were presented 4.8° off-center, to the left and to the right, in each trial.

#### Procedure

**Figure 1:**
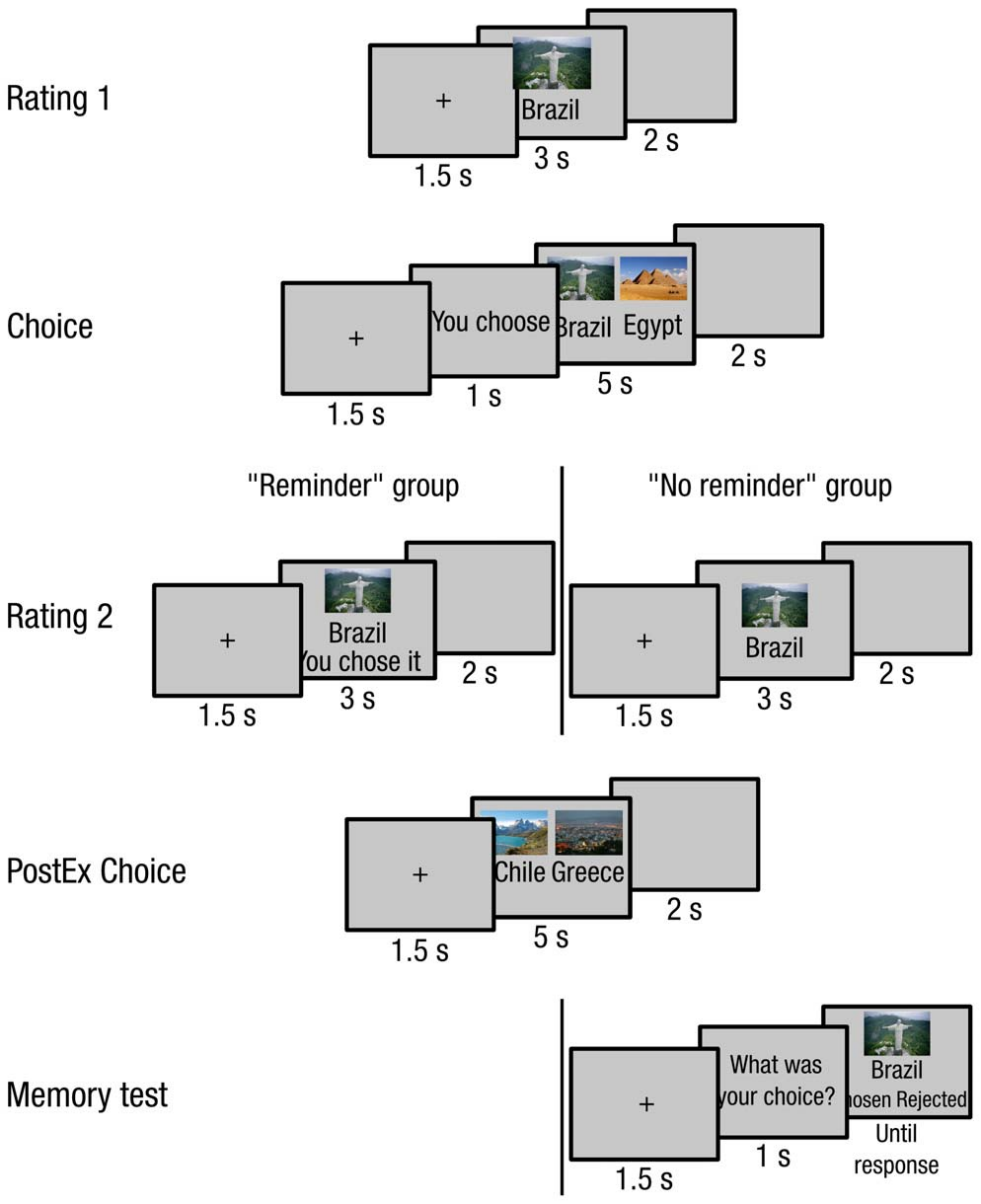
Experimental paradigm for the ‘Reminder’ and ‘No reminder’ groups. The experimental paradigm included 5 stages: Rating 1, Choice 1, Rating 2, Choice 2 and Memory test. The procedure differed between the ‘Reminder’ and ‘No reminder’ groups in Rating 2 and Memory test. In Rating 2, previous choice (either subject’s or computer) was indicated for the ‘Reminder’ group (left panel) but not for the ‘No reminder’ group (right panel). Additionally, subjects in the ‘No reminder’ group performed a memory test designed to check whether they have remember the information corresponding to the reminder present in Rating 2 for the ‘Reminder’ group.

In the ‘Reminder’ group, we followed a procedure similar to the one reported by Izuma et al. (2010) [16]. The experiment was composed of five blocks: two ‘Rating’ blocks (Rating 1 and 2) and two ‘Choice’ blocks (Choice 1 and PostEx Choice) and a ‘Memory’ block for the ‘No Reminder’ group (Figure 1).

The experiment started with ‘Rating 1′ block, which included 120 trials. Each trial began with a fixation point presented during 1.5-seconds. Then one vacation destination was centrally presented for 3 seconds, and followed by a blank screen lasting for 2 seconds during which subjects were requested to report how much they would like to spend their vacation in this destination using an eight-point scale (1 = ‘I do not want to go there at all’, 8 = ‘I definitely want to go there’). Subjects responded using the 1–8 number pad buttons of a regular keyboard. In the absence of response, the experiment proceeded to the next trial.

This first block was followed by ‘Choice 1′ block. In this block, every trial started with a fixation point presented during 1.5-second. Then a task instruction appeared for 1 second, indicating whether the subject should choose between the two destinations to be proposed on the next screen (‘You choose’), or whether he should observe computer’s choice (‘Computer chooses’). Then, two destinations were presented side-by-side for 5 seconds. Only in the ‘computer chooses’ trials a black frame surrounded the chosen destination. Trials ended with a 2-second presentation of a blank screen. Subjects had to report manually either computer’s choice or their own choice using the left/right arrows keyboard keys. Crucially, destinations were coupled according to subjects’ responses in ‘Rating 1′ block, as to create 3 experimental conditions: ‘Self-Easy’, ‘Self-Difficult’ and ‘Computer’. ‘Self-Easy’ trials were composed of a highly rated destination (rating ≥5) and a low rated destination (rating <5) that differed in rating by at least 3 points. ‘Self-Difficult’ and ‘Computer’ trials were composed of two highly rated destinations (rating ≥5) that differed in rating by no more than one point. The number of couples for each sequence was equal, but changed from subject to subject (from 8–19 for each sequence).

The third experimental stage was ‘Rating 2′ block, and differed between the ‘Reminder’ and ‘No reminder’ groups. In the ‘No reminder’ group, this block was strictly identical to ‘Rating 1′. In the ‘Reminder’ group, a written reminder indicating subject’s or computer’s choice concerning this item during ‘Choice 1′ block (e.g.: ‘you chose it’ or ‘computer rejected it’) was added. As in Izuma et al. (2010), subjects were instructed to ignore these comments.

This ‘Rating 2′ block was followed by ‘PostEx Choice’ block, in which only couples of destinations used in the ‘Computer’ condition during ‘Choice 1′ block were presented. Subjects had to choose between these items, as described for ‘Choice 1′ block.

Finally, a memory test was added at the end of the experiment for the ‘No reminder’ group. Every trial began by an instruction presented for 1 second and specifying which event should be recalled on the following destination: either subject’s own choice or computer’s choice concerning this item in ‘Choice 1′ block. Note that this memory test was addressing the information reminded to subjects during ‘Rating 2′ in the ‘Reminder’ group. Importantly, subjects were not informed about the existence of this final memory test when they performed the first 4 blocks, so they were not explicitly instructed to memorize their choices.

As we were interested in testing whether subjects remembered their choices, we considered items as remembered only if subjects correctly reported whether they had chosen or rejected each of the two coupled items.

## Results

**Figure 2:**
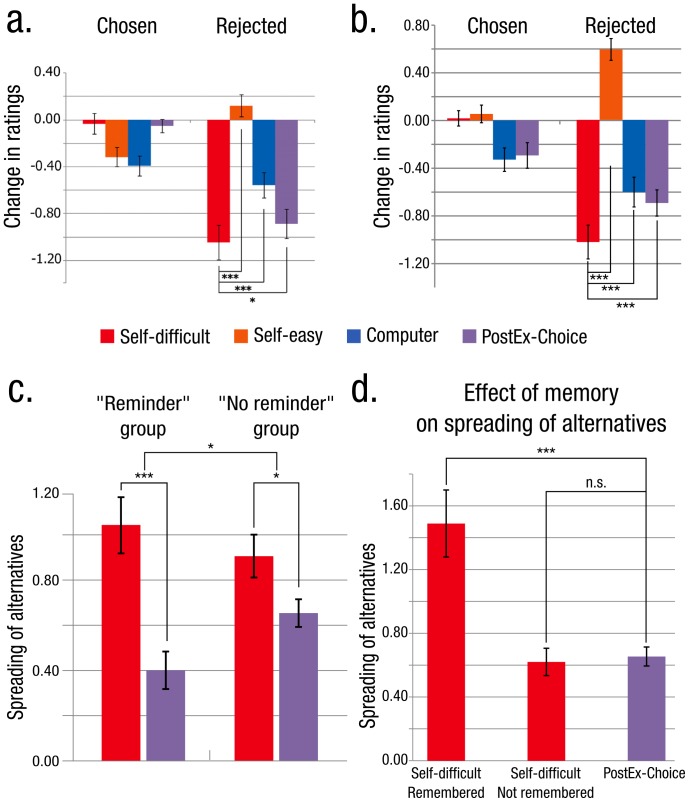
Spreading of alternatives in experiment 1. (**a**) Results obtained by Izuma et al. 2010 (adapted from PNAS). Bars represent the change in ratings between Rating 2 and Rating 1 for chosen items (left) and rejected items (right) in each condition. The critical comparison (corresponding to the comparison between RCR and RRC sequences respectively) is between Self-Difficult and PostEx-Choice. * p<0.05, *** p<0.001 (paired t-test, one tailed). (**b**) Replication of Izuma et al’s experiment (‘Reminder’ group). *** p<0.001 (paired t-test, one tailed). (**c**) Spreading of alternatives in the ‘Reminder’ (left) and ‘No reminder’ (right) groups. The interaction between the sequence (RCR – Self-difficult/RRC – PostEx-Choice) is significant (* p<0.05, ANOVA) and the critical comparison between RCR - Self-difficult and RRC - PostEx-Choice conditions is significant in both groups (* p<0.05, *** p<0.001, paired t test, two tailed).(**d**) Effect of memory on spreading of alternatives. The difference between the spread in the RCR – Self-difficult and the RRC – PostEx-Choice was significant only in pairs for which the choice was remembered (*** p<0.001, paired t test, two tailed).

We first replicated results reported by Izuma et al ([16] - see Figure 2a and 2b). Indeed, within the ‘Reminder’ group which is a direct adaptation of Izuma et al.’s procedure to our vacation destinations dataset, preference for destinations which were rejected in the Self-Difficult condition significantly decreased compared with rejected destinations in the Self-Easy condition (t(23) = 9.67, p<10^−8^) or those rejected in the Computer condition (t(23) = 4.14, p<0.001). These comparisons were used before Chen and Risen’s criticism to assess the existence of preference change, but they do not allow controlling for the statistical artefact highlighted by Chen and Risen [18]. Importantly, in this group, we also observed a significant difference between the critical condition and the proper control condition: the spread was significantly larger for the Self-difficult/RCR sequence than for the PostEx-Choice/RRC sequence (t(23) = 5.1, p<0.001). This replicates the critical behavioral result reported by Izuma et al [16].

We then focus on the two critical conditions: the Self-Difficult/RCR condition and the Post-Ex Choice/RRC condition, as the Self-Easy and the Computer are control conditions which do not take into account Chen and Risen’s criticism. We ran an analysis of variance (ANOVA) on the spread observed after difficult choices with an inter-subject factor (‘Reminder’/‘No reminder’) and a within-subject sequence factor (Self-Difficult RCR/Post-Ex Choice RRC). The reminder factor was not found significant (F<1). A main effect of sequence was observed (F(1,92) = 28.5; p<0.001) with larger spread for RCR than for RRC sequence. Crucially, these two factors interacted (F(1,92) = 5.5; p = 0.02) with larger difference in spread between RCR and RRC sequences in the ‘Reminder’ group than in the ‘No reminder’ group (mean difference in spread between RCR and RRC sequence for ‘Reminder’ group: 0.64, SEM = 0.13, for ‘No reminder’ group: 0.25, SEM = 0.11). Restricted analyses showed that in each of the two groups, spread was significantly larger for RCR than for RRC (for the ‘Reminder’ group: t(23) = 5.1; p<0.001 and for the ‘No reminder’ group (t(23) = 2.3, p = 0.03). Taken together these results suggest the existence of genuine choice-induced preference change, even after taking into account both the statistical flaw revealed by Chen and Risen [18] and the confound introduced by the reminder. They also reveal the strong impact of the reminder, suggesting that the availability of choice information during the second rating enhances preference change.

In order to refine this new finding, we focused on the ‘No reminder’ group in which choice-information was not delivered during the second rating. Overall, while subjects were at chance for remembering computer’ choices, they were still above chance for their own choices (t(23) = 9.87, p<0.001), leaving open the possibility that the availability of choice information during the second rating was modulating the spread. We then categorized each item as correctly or incorrectly memorized using the memory test performance. Crucially, remembered RCR trials showed a larger spread than RRC trials (t(23) = 3.72, p = 0.001), whereas no such a difference could be observed on forgotten RCR trials (t <1).

Taken together these results reveal the importance of the availability of choice information in the magnitude of preference change effects, be it through an external reminder or a spontaneous recall of the choice.

## Experiment 2

We designed and ran a second experiment with the following four objectives.

First, we aimed at replicating in a new group of subjects the effect of choice memory on the spread.

Second we modified the memory task in order to be able to cross RCR/RRC sequences with remembered/forgotten status of individual items, and therefore to test for the existence of an interaction between these two factors. Indeed, in the ‘No reminder’ group of the first experiment, the memory test was designed to probe the availability of information related to the reminder used by Izuma et al. (2010) [16]. Therefore, the memory test probed subjects’ and computer’s choices from the first choice block, but not subjects’ choices from the second choice block. So, this design could not allow us to properly cross memory and RCR/RRC factors, given that we did not have memory information on the choices made in the RRC sequence.

Third, one issue raised by our findings relates to the timing of preference change. Does it happen during the second rating, or could it occur immediately after the choice? As is, our memory test does not allow us to solve this issue given that a late recall of the choice is not diagnostic of the dynamics of preference change. In order to address this question, we compared spread values between blocks similar to the ‘No reminder’ group of experiment 1, and blocks during which subjects had to perform a demanding 2-back task immediately after their choices. This manipulation aimed at interfering with working memory and conscious elaboration about subjects’ own preferences during the choice.

Finally, we also aimed at disentangling memory from subjective relevance. Indeed, memory performance observed in experiment 1 could potentially be a side-effect of subjective relevance: subjects remembering their favorite or highly-rated choices better. Under this hypothesis, memory would not play a direct role in preference change. To address this issue, we coupled equally rated items from the whole rating scale and not only from the high rating values as we did in experiment 1.

### Methods

#### Participants

Thirty nine subjects (22 women; age M = 22.9 years old, SD = 3.1; 95% right-handed) participated in this study (see Ethics Statement above). All reported normal or corrected-to-normal visual acuity. The data from 3 participants were excluded from the analysis. These subjects were removed because their responses were shifted toward scale’s extremities (‘Rating 1’ median = 8 for two of them and 1 for the other).

#### Stimuli

Stimuli were the same images of vacation destinations as in the experiment 1. In the n-back task, stimuli were capital letters (size 18) presented at fixation for 1 second.

#### Procedure

**Figure 3:**
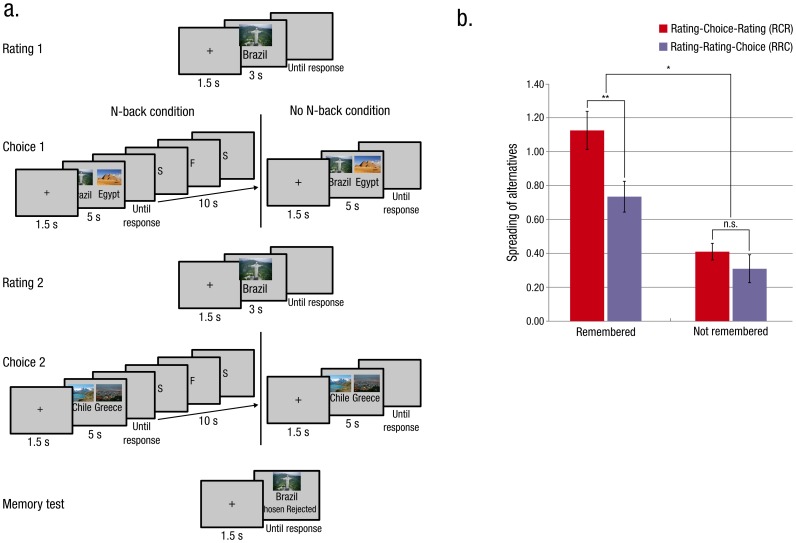
Experimental paradigm and effect of memory in Experiment 2. (**a**) Experimental paradigm for Experiment 2. Five stages were included: Rating 1, Choice 1, Rating 2, Choice 2 and Memory test. Each ‘Choice’ block comprised two sub-blocks: one in which the choice was immediately followed by a 2-back task and one including only the choice.(**b**) Effect of memory on spreading of alternatives. The interaction between the sequence (RCR/RRC) and the memorization of the choices was significant (* p = 0.019). The difference between the spread in the RCR and the RRC sequences was significant only in pairs for which the choice was remembered (** p<0.01, paired t test, two tailed).

Procedure was similar to the one used in the first experiment with four major differences (see Figure 3a). First, subjects’ responses were not limited in time. Second, items were coupled by sorting them according to their first rating resulting in 60 pairs of similarly rated items. Third, each ‘Choice’ block was divided into 2 sub-blocks composed of 15 couples of items. Pairs of items were randomly attributed to one of these sub-blocks. The first sub-block was identical to the ’Choice 1’ block in the previous experiment. In the second sub-block, subjects had to perform a 2-back task after each choice. Ten letters were presented at fixation, and subjects had to report a letter repetition if it was separated by two other letters by pressing the spacebar key. Fourth, in the memory test, subjects had to report whether they had chosen or rejected the item during the first or the second ‘Choice’ block.

## Results

We first tested the effect of the manipulation we used by an ANOVA on spread including sequence (RCR/RRC) and manipulation (N-back/No N-back) as factors. A main effect of sequence was found (F(1,140) = 5.54, p = 0.02). The main effect of manipulation almost reached significance (F(1,140) = 3.68, p = 0.06). But the interaction between sequence and manipulation was not significant (F<1).

Memory-test scores showed that the N-back manipulation was effective as choices that were followed by it were remembered less than those that were not (mean N-back: 0.32, SEM = 0.02, No N-back: 0.44, SEM = 0.03; t(35) = 4.95 p<0.001).

As we were mainly interested in the relationship between memory and spread we also tested the effect of memory in this task using a linear mixed-effects model including the spread for each pair of items for each subject, with the ‘Sequence’ (RCR/RRC) and the ‘Memory’ (remembered/forgotten) factors defined as fixed factors and subjects defined as a random factor. Indeed, as opposed to a classical ANOVA, such a model does not require prior averaging of the spread for each subject and thus offer the possibility to handle the heteroskedasticity related to the unbalanced number of items in each condition [28]. Significance of the fixed effects was assessed using the Kenward-Roger approximation for degrees of freedom of the denominator, with the ‘lmerTest’ package in R. A main effect of memory was found (F(1,2112) = 64.7, p<10^−10^), as well as a main effect of sequence (F(1,2124.3) = 15.7, p<10^−4^). The critical interaction between memory and sequence was significant (F(1,2145) = 5.5, p = 0.019). Indeed the difference between RCR and RRC was highly significant for remembered items (F(1,795.6) = 15.3, p<10^−4^), but was not significant for forgotten items (F(1,1325.4) = 1.68, p = 0.195). Whereas we observed, as we expected, a larger spread for remembered than for forgotten items in the RCR sequence (F(1,1021.9) = 53.4, p<10^−10^), the same effect was surprisingly also found in the RRC sequence (F(1, 1076) = 17.0, p<10^−4^).

## Discussion

In this study, we obtained four results that will structure our discussion.

First, we confirmed the existence of the artifact discovered by Chen and Risen [18]. Indeed, spreads obtained in the RRC control sequence were systematically significantly larger than zero. This confirms Chen and Risen’ argument that choices hold information about preferences and that they should not be only considered as a source of preference change.

Moreover, we found a significantly larger spread for items which were correctly remembered as chosen or rejected in the RRC sequence than for items which were not correctly remembered. Within the Chen & Risen framework, this unexpected effect could simply reflect the impact of a better attentional engagement during some choices, which would in turn reveal more accurate information about preferences. Indeed, given that memory encoding depends on attention (see [29] for a review), a choice that was later remembered was presumably processed with more attention than a choice that was later forgotten. Moreover, attended choices may well provide more information about subjects’ preferences than less attended choices. As a direct consequence of this bias and in agreement with our results, larger spread would be observed for memorized RRC items than for forgotten ones. This would also be reflected in an increased number of pairs in which spread was positive. Our results did show this pattern, as the mean proportion of couples for which positive spread was achieved in the RRC sequence was higher (t(35) = 2.51, p<0.02) for remembered choices (mean = 0.842, SEM = 0.020) than for forgotten choices (mean = 0.774, SEM = 0.025).

Second, in spite of the artifact pointed out by Chen and Risen, we replicated the finding reported by Izuma et al. [16], by observing a significantly larger spread in the RCR sequence than in the RRC sequence. This result, which we replicated in our two experiments, strengthens the existence of genuine spreading of alternatives in the FCP paradigm. To date, our current work counts among the rare robust results within an empirical literature weakened by the seminal study of Chen & Risen (see also [21], [23]).

Third, we showed that the reminder cue used by Izuma and colleagues [16] affected drastically the measured spread. As we mentioned above, Izuma et al. did not compare RCR versus RRC, but rather ‘RCR+explicit choice reminder’ versus RRC. We observed that in the absence of such a reminder cue, the difference between RCR and RRC spreads was much smaller than when a cue was used. This result is important in regard to the current theoretical debate about the precise mechanism at work in choice-induced preference change. According to models postulating automatic updating of values independent of episodic memory, such an explicit cue should not affect spread values. Alternatively, self-related models postulate that such episodic memory cue should decisively affect the spread by loading conscious working memory with choice information. Our results thus tend to favor this second hypothesis.

Finally and most importantly, we found a strong relation between choice memory and choice-induced preference change. Only remembered items showed a significantly larger spread for RCR than for RRC. We replicated this original finding across two experiments with different subjects and two different paradigms. It may explain, in part, the boosting effect of the reminder cue used by Izuma et al [16] on preference change. In experiment 1, we showed that in the absence of the reminder cue, a spread was observed in pairs of items, for which choice was remembered, and was absent in pairs for which choice was forgotten. In experiment 2, we tried to manipulate choice encoding by loading working memory and attentional resources, using a 2-back task immediately after choice in half of the trials. We succeeded only in part, as choices that were followed by the 2-back task were mostly forgotten, but choices that were not followed by the task were not all remembered. We then examined spreading of alternatives according to subjects’ performance on the memory test, irrespective of the post-choice manipulation. This analysis confirmed that the spread was larger for RCR sequence than for RRC sequence only if choices were remembered.

This correlation between spread and memory raises the issue of causality between episodic memory of choices and spread, and of the timing of observed preference changes. Is this correlation pointing to a causal link between episodic memory of choices and spread? One possible interpretation of our results, - which supports self-related theories -, posits that preference change occurs only when subjects are confronted to a self-coherence context such as the second rating (R2) for items which were correctly remembered as chosen or rejected. Under this assumption, preference change would not occur implicitly or unconsciously immediately after the choice. In other words, choice-induced preference change may actually be a ‘choice-rating consistency induced’ preference change occurring exclusively during the second rating. Alternatively, supporters of implicit theories of preference change may propose that memory of choices could be more accurate for items showing a large spread than for those with more stable ratings. Under this view, a reverse causality would be proposed: memory would not cause preference change, but preference change would enhance performance in the memory test. This would be coherent with the observation of significant spread in amnesic patients before Chen and Risen’s criticism ([13]). Such an enhancement of performance in the memory test could stem either from a direct increase of episodic memory or from a guessing bias: subjects would guess their previous choices by probing their current updated value of a given item to infer their choice. Both the episodic memory enhancement and the guessing strategy hypotheses share a common prediction: this effect should be item-specific and not related to pairs of items proposed during the choice. Thus, we should observe a larger mean absolute value of ratings variation (mean of |R2-R1|) for remembered than for forgotten items. We ran this analysis and observed no difference of this index between remembered vs forgotten at the single item level in the RCR (t(35) = −0.47; p = 0.6) and in the RRC (t(35) = −0.34; p = 0.34) sequences. Moreover, our criterion of correct memory performance (correct answers to both items presented during a choice episode) was more prone to target episodic memory of the whole choice than episodic memory of a single item or choice guessing because both items of a given choice had to be correctly answered to categorize the memory answer as correct.

Taking all these considerations together, our findings seem to better support self-related models of preference change, even if the correlation we observed between memory performance and spread is far from being a direct evidence of a causal link.

However, we should improve the memory test in a way to disentangle the respective roles of episodic memory and inferential guessing. For instance, a subjective report such as a “remember/know” or “remember/guess” task could be added in order to better disentangle between these two possibilities. Additionally, the use of physiological measures or functional brain imaging recordings in the same paradigm could be useful to determine if preference change occurs before or after the second rating.

Another related question stemming from our results concerns the nature of the preference change effect: is it only a transient representation elicited by a need of consistency between past and present, within a working-memory system, or is it rather durably encoded in subject’s value system? Future experiments could resolve these issues by examining the dynamics of preference change and the genuine impact of episodic memory on it. For instance, exploration of amnesic versus dysexecutive patients may help dissecting the role of episodic memory and executive functions in the processing of coherence at each experimental stage (choice and second rating). Functional brain-imaging could also be used to probe the respective contributions of episodic memory, executive control [12] and value networks [17] during both the choice and the second rating.

We conclude by stating that genuine choice-induced preference change effect does exist in the FCP, after controlling for two important confounds, and that it is related to episodic memory. Our results tend to support self-related models of preference change. But the precise role of conscious, - accessible to subjective reports -, and of non-conscious processes remain to be addressed in future studies.
